# Long-Term Consequences of Worsened Poststroke Status in Patients With Premorbid Disability: Implications for Treatment

**DOI:** 10.1161/STROKEAHA.118.022416

**Published:** 2018-09-10

**Authors:** Aravind Ganesh, Ramon Luengo-Fernandez, Sarah T. Pendlebury, Peter M. Rothwell

**Affiliations:** From the Nuffield Department of Clinical Neurosciences, Stroke Prevention Research Unit, University of Oxford, United Kingdom.

**Keywords:** cerebrovascular, disorders, cohort studies, humans, prognosis, survivors

## Abstract

**Background and Purpose—:**

Patients with premorbid disability, generally defined as modified Rankin Scale (mRS) score ≥2, are often excluded from trials of acute stroke therapies. However, increased disability in such patients will adversely affect long-term outcomes if treatments are withheld in routine practice. We assessed the extent to which increased disability poststroke influences 5-year mortality, institutionalization, and costs in premorbidly disabled patients.

**Methods—:**

In a population-based, prospective cohort of patients with ischemic stroke (OXVASC [Oxford Vascular Study], 2002–2014), we tracked mortality, institutionalization, and healthcare/social-care costs during follow-up. We compared 5-year mortality and poststroke institutionalization (Cox regressions) and 5-year healthcare/social-care costs (generalized linear model) in 3-month survivors with premorbid mRS of 2 to 4 (excluding extreme disability, mRS=5), based on the degree of change in mRS(ΔmRS) from prestroke to 3 months poststroke, adjusting analyses for age/sex/initial National Institutes of Health Stroke Scale.

**Results—:**

Among 1607 patients, 530 (33.0%) had premorbid mRS of 2 to 4. Only 2 premorbidly disabled patients received thrombolysis, but 421 (79.4%) were alive at 3 months. ΔmRS was independently associated with 5-year mortality/institutionalization (adjusted hazard ratio for ΔmRS=1 versus 0: 1.59; 95% CI, 1.20–2.11; ΔmRS=2: 2.39; 95% CI, 1.62–3.53; ΔmRS=3: 4.12; 95% CI, 1.98–8.60; *P*<0.001) and costs (margin for ΔmRS ≥2 versus 0: $30 011, 95% CI, $4222–55 801; *P*=0.023). Results were similar on examining patients with premorbid mRS of 2, 3, and 4 separately (eg, 5-year mortality/institutionalization adjusted hazard ratio for premorbid mRS=3 with ΔmRS=1 versus 0: 1.60; 95% CI, 1.06–2.42; *P*=0.027; ΔmRS=2: 3.20; 95% CI, 1.85–5.54; *P*<0.001).

**Conclusions—:**

Patients with stroke with premorbid disability have higher mortality, institutionalization, and costs if they accumulate additional disability because of the stroke. These findings highlight the long-term outcomes expected if acute interventions are routinely withheld in patients with mild-moderate premorbid disability and suggest that trials/registries should include such patients.

## Abstract 2

Supplemental Digital Content is available in the text.

Acute interventions, such as thrombolysis and thrombectomy, reduce disability in patients with ischemic stroke.^1,2^ However, evidence of benefit is lacking in patients with prestroke disability, who have generally been excluded from acute stroke trials. For instance, most thrombectomy trials excluded patients with premorbid modified Rankin Scale (mRS) score ≥2.^2^ This approach is not based on any mechanistic hypothesis about reduced benefit in such patients but simply reflects the fact that premorbid disability prevents such patients from contributing to typical dichotomy-based definitions of favorable outcome used in trials (eg, mRS, 0–1 or 0–2). Patients with mild-to-moderate disability (mRS, 2–4) would likely consider retaining their premorbid status as a favorable outcome, but premorbid disability is often cited as a reason for excluding patients from thrombolysis, despite not being a formal contraindication.^3^

Part of the reluctance to treat premorbidly disabled patients is their perceived poor prognosis, with registry data indicating a high inpatient mortality,^4^ and many acute stroke risk stratification scales using premorbid disability as a predictor.^5,6^ One study of sequential hospital admissions with acute stroke found that every point increase in prestroke mRS was associated with poorer outcomes (length-of-stay, discharge destination, mortality, complications), which were not explained by the influence of prestroke mRS on care pathways.^7^ Although poorer prognosis does not rule out clinically meaningful acute treatment effects, European guidelines recommend that patients selected for acute stroke interventions should have a prestroke mRS of 0 to 1, while indicating the absence of evidence to support specific recommendations in those with prestroke mRS ≥2.^8^ Based on a review of the scientific rationale for thrombolysis criteria in acute stroke,^9^ American guidelines state that premorbid disability does not seem to increase the risk of post-thrombolysis hemorrhage and that thrombolysis/thrombectomy may be reasonable in selected cases but also that treatment may be associated with less neurological improvement and higher mortality.^10^ Small studies of thrombolysis in premorbidly disabled patients indicate that they have a higher mortality risk but appear able to return to prestroke status as often as patients without preexisting disability.^11,12^ In addition, an analysis of 7250 patients with stroke in the Safe Implementation of Treatments in Stroke-Eastern Europe registry, in which 293 (4%) patients had a prestroke mRS of 2 and 171 (2%) had prestroke mRS ≥3, found no independent association between prestroke disability and the risk of symptomatic intracerebral hemorrhage post-thrombolysis and found that 1 in 3 previously disabled patients could return to their prestroke mRS, despite higher mortality.^13^

Use of acute therapies in premorbidly disabled patients would be further justified if increased poststroke disability could be shown to worsen other long-term outcomes if treatments are routinely withheld. We, therefore, determined the prevalence of premorbid disability and the extent to which increased poststroke disability influences 5-year mortality, institutionalization, and care costs in premorbidly disabled patients, in a population-based, prospective cohort study (OXVASC [Oxford Vascular Study]).

## Methods

The data that support the findings of this study are available from the corresponding author upon reasonable request.

The OXVASC population comprises 92 728 patients registered with about 100 general practitioners in 9 practices across Oxfordshire. Study methods have been published.^14^ Recruitment has been ongoing since April 2002. Near-complete ascertainment^15^ of suspected stroke/transient ischemic attack cases is achieved using overlapping methods of hot and cold pursuit, including daily rapid-access transient ischemic attack/stroke clinic to which participating general practitioners and the local emergency department (accident and emergency) refer all individuals with suspected but not hospitalized transient ischemic attack/stroke; daily searches of ward admissions (medical, cardiology, stroke, and neurology), accident and emergency attendance register, and in-hospital Bereavement Office death records; and monthly searches of death certificates, coroners’ reports (for out-of-hospital deaths), general practitioner and hospital diagnostic/discharge codes, and brain/vascular imaging referrals. Direct assessment has shown ascertainment is near complete.^15^

Patients with ischemic stroke recruited from April 2002 to March 2014 were included. Patients were assessed urgently by study clinicians and considered for inclusion. Informed consent was obtained from patients whenever possible; otherwise, assent was obtained from caregivers if patients were unable to consent. Stroke was diagnosed per the World Health Organization definition.^16^ Neurological impairment, medical history, and risk factors were assessed. Stroke severity was measured using the National Institutes of Health Stroke Scale (NIHSS). All cases were reviewed with senior neurologist P.M.R. daily and imaging reviewed by the study neuroradiologist.

Premorbid disability was assessed using the mRS as part of the initial patient assessment, performed as soon as possible after the stroke. Prestroke mRS has been shown to be a moderately valid measure of prestroke disability and a robust predictor of poststroke prognosis.^7,17^ All mRS assessments were performed by clinical staff using a structured interview and based on history taken directly from patients whenever possible, supplemented by review of primary care and hospital medical records and interviews with relatives and other informants. The 20-point Barthel index was also used as a supplementary measure of ability to perform activities of daily living.^18^ Patients were classified as socioeconomically deprived if their index of socioeconomic deprivation was worse than the median for the study population.

Patients had face-to-face follow-up with a study nurse/physician either in a hospital clinic or at home at 1 month, 3 months, 6 months, 1 year, and 5 years. Recurrent vascular events and disability (mRS) were recorded at each follow-up. Raters were all trained in the use of the mRS using an instructional DVD with accompanying written materials produced by the University of Glasgow that has been used in large-scale clinical trials.^19^ At follow-up, patients and carers were asked about their living arrangements. In addition, patients’ hospital and general practitioner records were reviewed to identify whether and when they were institutionalized. Long-term institutionalization was defined as admission into a nursing or residential care home. We did not include temporary postacute care and in-hospital rehabilitation stays.

Patients who moved out of study area were followed up by telephone. Additional information was obtained from carers in patients with impaired cognition or speech. All deaths were recorded via death certificates, coroners’ reports, and the National Health Service Central Register. Healthcare and social-care resource use was obtained from the date of the first stroke in study period (index stroke) until 5 years poststroke. The methods on collection of resource use and costs have been reported previously.^20^ Briefly, patients’ records from the Oxford University Hospitals Trust were reviewed for any emergency visit/transport, outpatient care visit, day case, or hospitalization. For each spell in hospital, the dates of admission, discharge, and interward transfers were recorded. We estimated institutionalized days as the difference between either date of 5-year follow-up or death, whichever was the earliest, and date of admission into the institution. Hospital resource use was valued using unit costs from the National Health Service schedule of reference costs.^21^ Institutionalization was costed as the cost per week in a private nursing home, £795 ($1145) in 2016.^22^ All costs were presented in 2016 prices and converted from UK pounds sterling (£) to US dollars ($) using the 2016 rate of purchasing power parities ($=£0.694; http://stats.oecd.org/).

The study was approved by the Oxfordshire Research Ethics Committee.

### Statistical Analyses

Analyses were censored at May 15, 2017, and were performed using STATA 13.1. Summary statistics were used to examine the subset of all patients with ischemic stroke who had mild-to-moderate premorbid disability 2–4 and their disability/mortality outcome at 3 months. Cox regressions, adjusted for age, sex, and stroke severity (initial NIHSS score), were used to compare 5-year mortality or new poststroke institutionalization outcomes in 3-month survivors with premorbid mRS of 2 to 4, based on the degree of change in mRS (ΔmRS) from prestroke to 3 months poststroke. Patients with premorbid mRS of 5 were excluded from our analyses because they could not by definition demonstrate a nonzero ΔmRS if alive at 3 months because the next level up (mRS=6) is death. χ^2^ tests were used to compare proportions and the Wilcoxon rank-sum to compare continuous or pseudocontinuous variables.

We examined the effect of censoring on costs,^23^ partitioning the study period into smaller time periods (by day) within each of which the total cost incurred for all patients alive at the beginning of the period was calculated. Estimated costs of patients with complete data for each time period were weighted by the Kaplan-Meier sample average estimator and summed over all periods to estimate mean 5-year censor-adjusted costs. To assess whether healthcare/social-care costs varied over time by ΔmRS, we then constructed generalized gamma linear models assuming a log identity, adjusted for age, sex, and NIHSS.

Statistical significance was set at *P* <0.050.

## Results

**Figure 1.**
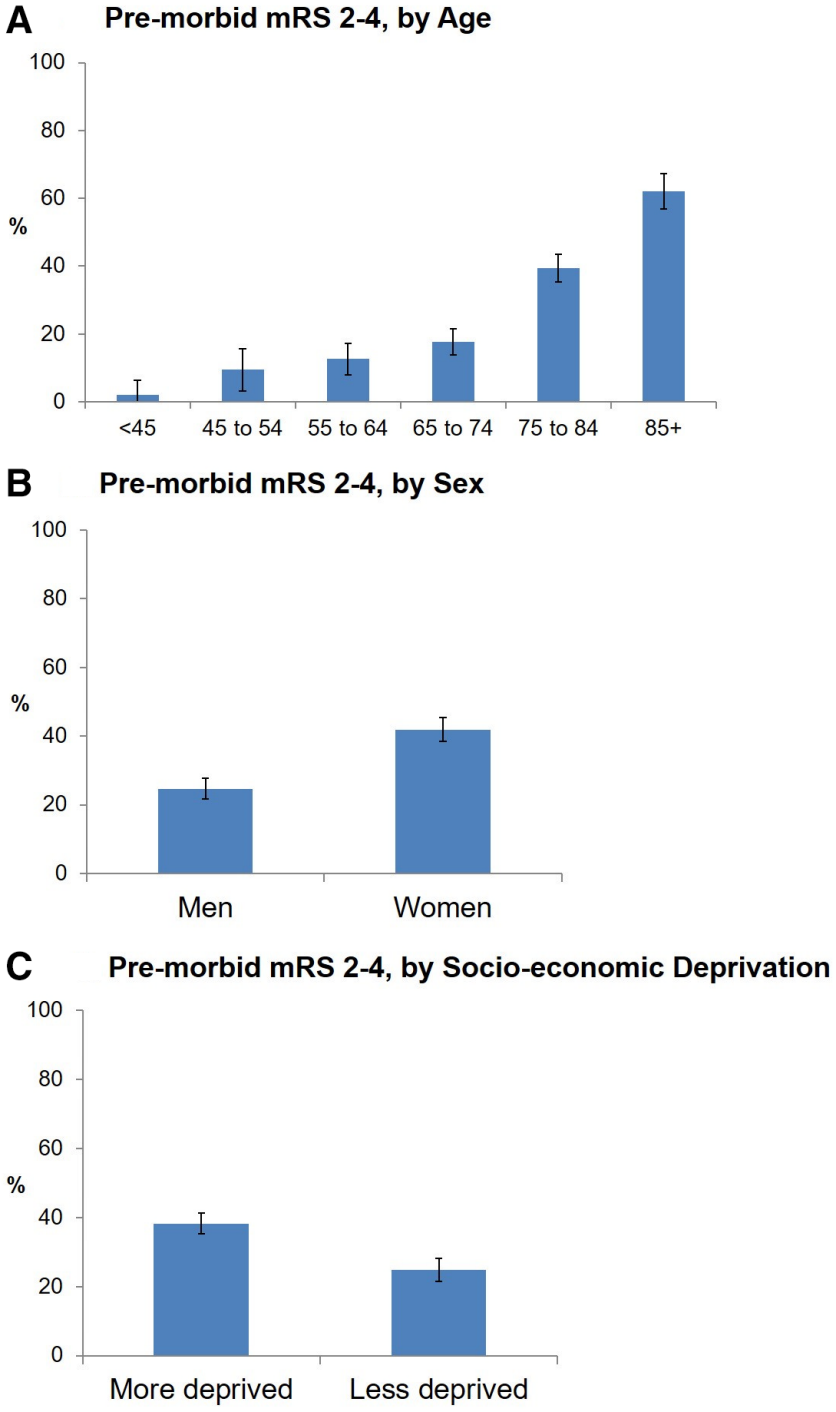
Proportion of patients with ischemic stroke with mild-to-moderate premorbid disability. This was defined as a prestroke mRS of 2-4 and is presented by: (**A**) age, (**B**) sex, and (**C**) socioeconomic deprivation index. Bars represent 95% CIs. mRS indicates modified Rankin Scale.

Among 1607 patients with ischemic stroke, 530 (33.0%) had premorbid mRS of 2 to 4 (18 had mRS=5). Premorbid mRS and premorbid Barthel index were strongly correlated (Spearman ρ, −0.59; mean Barthel index, 19.8; 95% CI, 19.8–19.9 for premorbid mRS 0–1, versus mean Barthel index, 17.4; 95% CI, 17.0–17.7 for premorbid mRS 2–4; *P*<0.0001). The proportion of patients with premorbid mRS of 2 to 4 rose with age and was higher among women than in men and among more socioeconomically deprived patients (Figure 1). Age, sex, and socioeconomic deprivation remained significantly associated with premorbid mRS of 2 to 4 on adjusted logistic regression (Table I in the online-only Data Supplement). Most of the premorbid disability was not stroke related because only 90 (17.0%) of those with premorbid mRS of 2 to 4 had a history of stroke. Only 2 premorbidly disabled patients received thrombolysis.

Compared with patients with premorbid mRS of 0 to 1, those with premorbid mRS of 2 to 4 were older, more often women (*P*<0.0001 for both), and more likely to have a history of stroke, myocardial infarction, atrial fibrillation, and diabetes mellitus, and be socioeconomically deprived and institutionalized prestroke (Table 1). The median NIHSS at presentation for the cohort was 2 (interquartile range, 1–6). Patients with premorbid mRS of 2 to 4 had more severe strokes, accounting for 212 (43.6%) of 486 major strokes (NIHSS, ≥5). They were more likely to be admitted to a stroke unit overall (47.5% versus 40.3%; *P*=0.002), but on adjusting for NIHSS, no difference was seen (aOR, 1.01; 95% CI, 0.80–1.29; *P*=0.91). Premorbidly disabled patients had longer lengths of stay (median, 27 versus 14 days; *P*=0.0001). Four hundred twenty-one (79.4%) patients with premorbid mRS of 2 to 4 were alive at 3 months; 3-month mRS was available for 410 (97.4%). Although 3-month mortality was higher for premorbidly disabled patients versus those with prestroke mRS of 0 to 1, they were less likely to have further poststroke disability (ΔmRS, ≥1) at 3 months (Table 1). Overall, 175 of 410 (42.7%) premorbidly disabled patients were left with ΔmRS ≥1; this proportion increased to 96 of 130 (73.9%) for major strokes. By 5 years poststroke, 350 (69.9%) of premorbidly disabled patients were dead, but these patients lived an average of 1.35 years (95% CI, 1.20–1.51) after their stroke. One hundred nineteen of 387 (30.8%) previously community-dwelling 3-month survivors required new institutionalization. Twenty-nine 3-month survivors (6.9%) had not yet reached 5 years poststroke, but all had data ≤3 years.

**Table 1.**
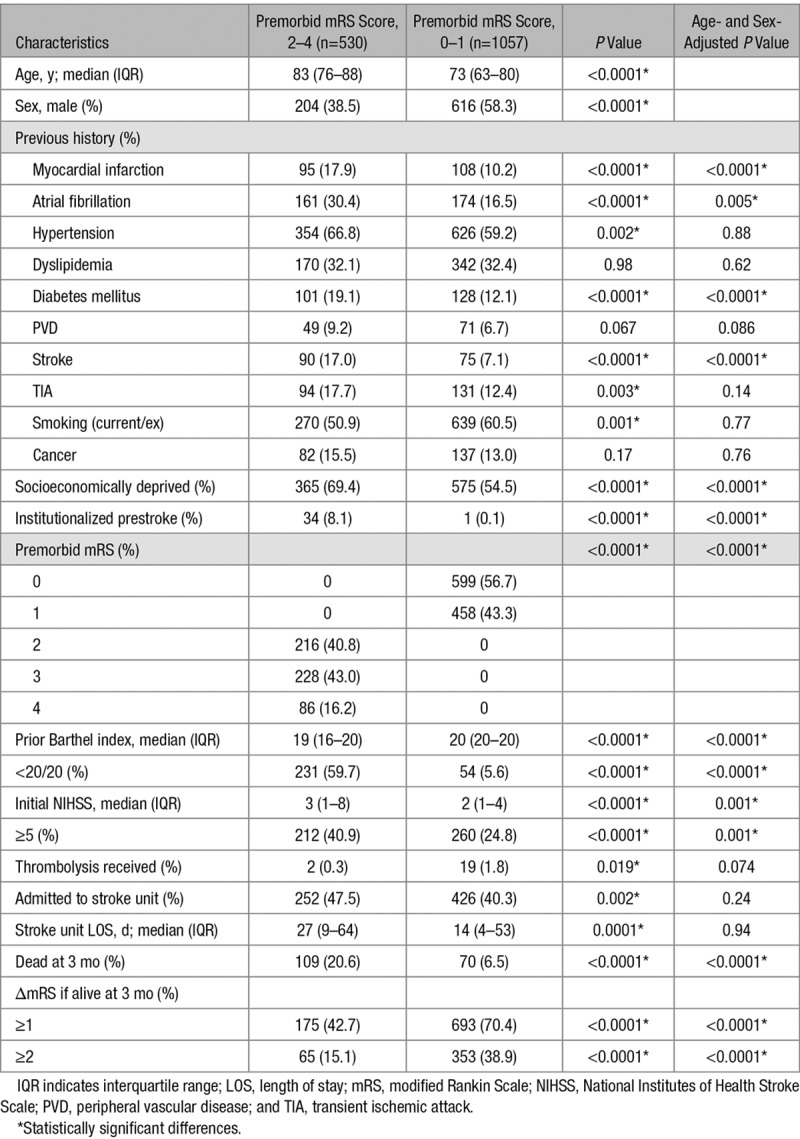
Patient Sample and Characteristics for Patients With Ischemic Stroke (n=1607), Excluding Patients With Prestroke mRS of 5 (n=18)

**Figure 2.**
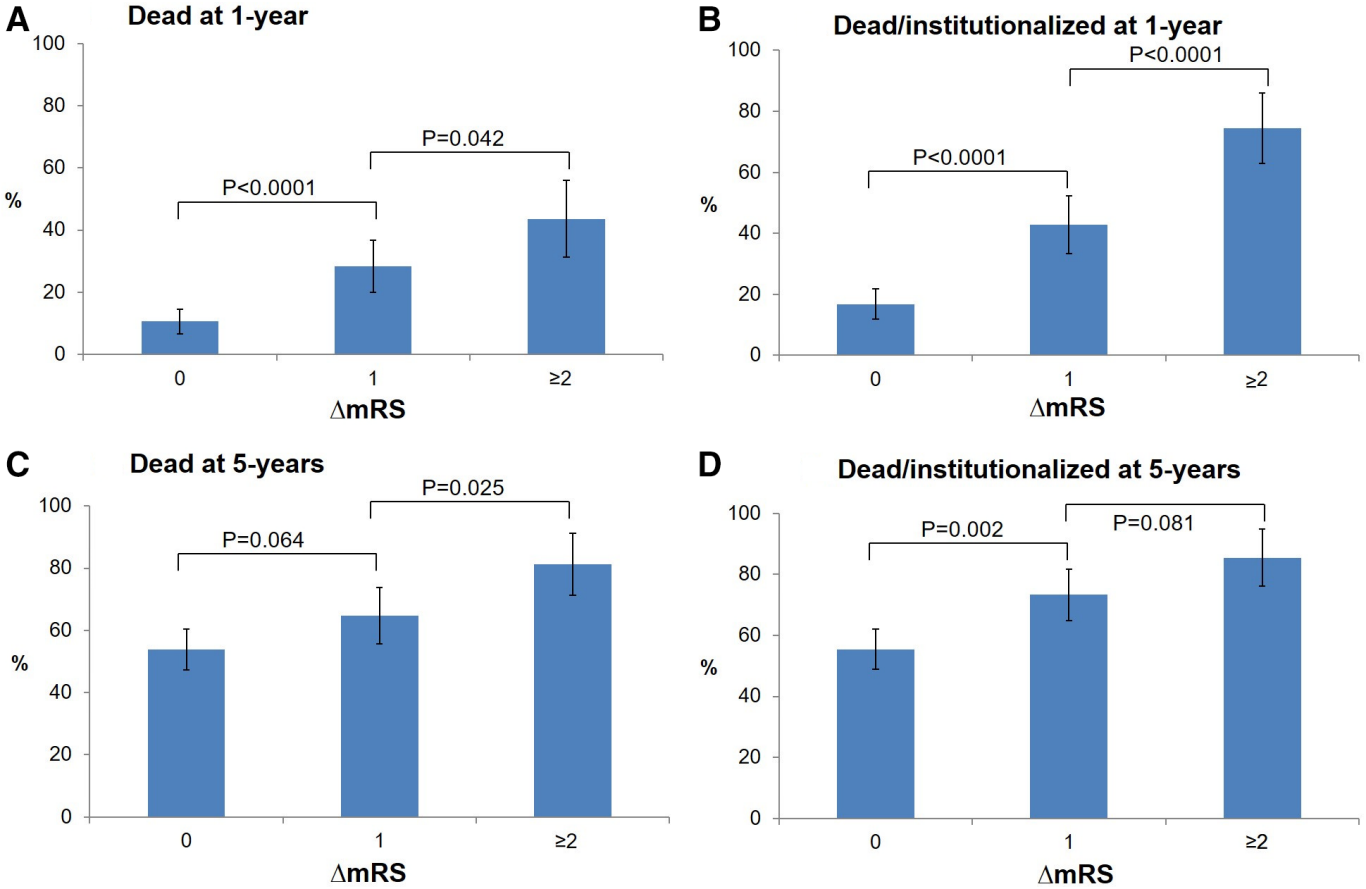
One- and 5-y outcomes of death and death/institutionalization in survivors of ischemic stroke with premorbid modified Rankin Scale (mRS) of 2 to 4, stratified by the change in mRS between prestroke and 3 mo poststroke (ΔmRS). **A**–**D**, The graphs show the proportion of 3-mo survivors, also alive at 1 y, who were (**A**) dead and (**B**) dead/institutionalized by 1 y poststroke and the proportion of 3-mo survivors also alive at 5 y, who were (**C**) dead and (**D**) dead/institutionalized by 5 y poststroke. Institutionalization was defined as admission to a nursing or residential care home. The *P* values from χ^2^ tests for differences between mRS grades are indicated. Bars represent 95% CIs.

ΔmRS was independently related to 1- and 5-year mortality and 5-year institutionalization (Figure 2; Figure I in the online-only Data Supplement), with each added degree of poststroke disability having worse outcome (eg, adjusted hazard ratio for 5-year mortality/institutionalization for ΔmRS=1 versus 0: 1.62; 95% CI, 1.21–2.18; ΔmRS=2: 2.11; 95% CI, 1.36–3.27; ΔmRS=3: 5.45; 95% CI, 2.58–11.5; *P*≤0.001; Table 2). Results were similar on examining premorbid mRS of 2, 3, and 4 separately (eg, 5-year mortality/institutionalization adjusted hazard ratio for premorbid mRS=3 with ΔmRS=1: 1.88; 95% CI, 1.21–2.93; *P*=0.005; ΔmRS=2: 3.36; 95% CI, 1.57–7.19; *P*=0.002; Tables II through IV in the online-only Data Supplement). The survival curves for both overall survival and institutionalization-free survival showed an early separation when premorbidly disabled patients were stratified by ΔmRS, well before 1-year follow-up, that was maintained at 5-year follow-up (*P*<0.0001; Figure II in the online-only Data Supplement).

**Table 2.**
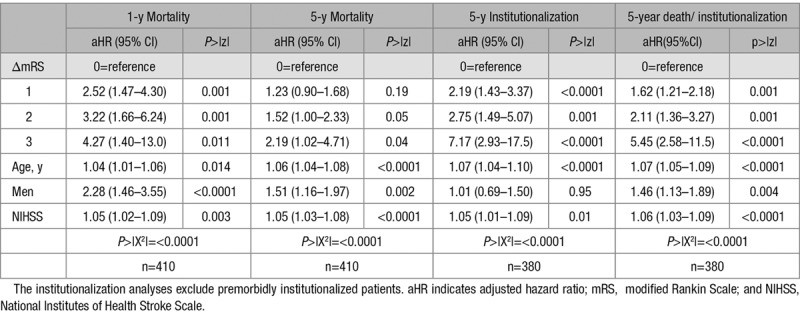
Impact of the Change in mRS From Prestroke to 3 Months Poststroke (ΔmRS) on 1- and 5-Year Mortality, Institutionalization, and Death/Institutionalization for 3-Month Survivors of Ischemic Stroke With Premorbid mRS of 2 to 4—Controlling for Age, Sex, and NIHSS Score at Acute Presentation

ΔmRS was also associated with 5-year healthcare and social-care costs, with the costs generally showing an upward trend with increasing ΔmRS, particularly when examining patients with premorbid mRS of 2 (Table V in the online-only Data Supplement). In particular, ΔmRS ≥2 was associated with a significant rise in 5-year costs in gamma linear model regression, adjusted for age/sex/NIHSS (margin versus mRS 0: $30 011; 95% CI, $4222–55 801; *P*=0.023), especially in those with premorbid mRS of 2 (margin versus mRS=0: $58 039; 95% CI, $16 414–99 663; *P*=0.006). The difference was even more marked upon excluding patients who were institutionalized prestroke or had not reached 5-year follow-up (margin for ΔmRS≥2 versus mRS=0, overall: $40 533; 95% CI, $8827–72 240; *P*=0.012; Table VI in the online-only Data Supplement).

## Discussion

In this population-based, prospective cohort study of 3-month survivors of ischemic stroke, we found that about a third of patients had mild-to-moderate premorbid disability (mRS of 2–4 before their stroke). We showed that if such patients accumulated additional disability as a result of their stroke, they had far worse 5-year mortality and institutionalization outcomes, and higher 5-year health/social-care costs, than those who retained their premorbid disability. These findings have implications for the management of patients with stroke with premorbid disability and for future trials of stroke therapies.

First, our findings highlight the substantial proportion of patients with stroke who would be deemed ineligible for acute stroke therapies if premorbid disability is routinely applied as an exclusion criterion. A third of our population had premorbid mRS of 2 to 4, and most survived a year or more poststroke, with >40% being left with further disability or requiring new institutionalization. Our analyses of 5-year care costs also highlight the additional long-term health economic burden associated with further poststroke disability in such patients. These data complement previous studies that have demonstrated the association of stroke severity or poststroke mRS with poststroke care costs,^20,24^ by separately examining the influence of incremental poststroke disability in premorbidly disabled patients. There is a pressing need for therapies that mitigate the burden of stroke in this population that is poorly served by current trial-based evidence.

Second, by demonstrating worse long-term outcomes with each additional disability increment poststroke in premorbidly disabled patients, our results suggest a role for therapies like thrombolysis and thrombectomy. Pursuing phase IV trials of approved acute stroke therapies in this population could help demonstrate treatment efficacy, but additional randomized controlled trials of thrombolysis/thrombectomy may not be feasible. Clinicians may be uncomfortable randomizing premorbidly disabled patients, particularly those with premorbid mRS of 2, into a noninterventional arm, questioning the ethics or equipoise of such trials. That being said, clinical decisions need not be informed by randomized controlled trials alone. For example, elderly patients were once considered ineligible for intravenous thrombolysis owing to trial-based contraindications in drug labels (eg, upper age limit in the ECASS [European Cooperative Acute Stroke Study] trials was 80 years),^25^ but this was later rejected by the stroke community after evidence from international registry-based studies like SITS (Safe Implementation of Thrombolysis in Stroke) demonstrated that the elderly can attain better outcomes with timely and judicious thrombolysis.^26,27^ Similarly, there is preliminary evidence from small observational studies that patients with premorbid disability can retain their premorbid status as often as premorbidly nondisabled patients.^10,11^ Similar results have been reported by observational studies that examined thrombolysis in patients with dementia.^28,29^ However, broader inclusion criteria in randomized trials would provide the most reliable evidence. Indeed, for thrombolysis in the elderly, it was the third IST (International Stroke Trial-3) that ultimately validated previous registry-based findings.^30^

Third, our findings suggest that the change in mRS from prestroke to poststroke (ΔmRS) may be a meaningful outcome measure in future trials that enroll a mix of patients with and without prestroke disability. We found that ΔmRS in premorbidly disabled patients was strongly associated with 5-year death, institutionalization, and healthcare costs, similar to the previously demonstrated worsening of long-term outcomes with each step up the 3-month mRS in the general stroke population.^31^ Currently, even if trials use ordinal analysis of the 3-month mRS, comparing differences across the range of the scale between treatment and control groups, enrolling patients with premorbid disability will lead to practical difficulties of adjusting for different levels of premorbid disability. Using a comparative measure like ΔmRS can mitigate this issue. However, the ΔmRS is limited by the fact that increments in mRS likely carry nonlinear differences in disability burden and prognosis: in other words, progression from mRS 2 to 3 is not equivalent to progression from 3 to 4 or from 4 to 5, despite each transition involving ΔmRS of 1.^32^ Furthermore, higher grades like mRS 3 or 4 account for a wide range of disabilities, so even if patients do not progress to the next mRS grade, this does not mean that their ability to perform everyday activities is unaffected.

Our analysis has several strengths, including a robust population-based design, high rates of ascertainment of incident strokes, completeness of follow-up, replication of findings for several outcomes, and generalizability, with similar 5-year mortality and institutionalization rates as prior population-based studies.^33,34^ The distribution of initial NIHSS scores in our study population is comparable with that reported in other population-based studies. For example, both the Cincinatti/Northern Kentucky Stroke Study and the Brain Attack Surveillance in Corpus Christi Project reported median NIHSS scores of 3 among 2233 and 1796 patients with stroke, respectively (the former additionally reported an interquartile range of 1–7), as compared with 2 (interquartile range, 1–6) in our study.^35,36^ NIHSS scores were lower in patients with premorbid mRS of 0 to 1 in our study (median, 2; interquartile range, 1–4); however, the aforementioned studies did not stratify initial NIHSS by premorbid mRS. Nevertheless, there are some potential shortcomings. First, there is potential for interrater variability in mRS.^37^ However, we sought to mitigate inaccuracies in prestroke and poststroke mRS using structured interviews by trained staff and corroborating information from multiple sources, including the patient (whenever possible), carers/family, and medical records. Moreover, our data reflect how premorbid and poststroke status are assessed in clinical practice and in trials. Second, we combined patients with premorbid mRS of 2 to 4 in our main analyses, but this reflects a broad spectrum of disability, and in practice, clinicians are likely to be more comfortable providing acute stroke therapies to functionally independent patients (mRS=2) than those with mRS of 3 to 4. However, we also found significant increases in 5-year mortality/institutionalization with increasing ΔmRS on examining patients with premorbid mRS of 2, 3, and 4 separately. Third, thrombolysis rates were relatively low in our cohort even for patients with premorbid mRS of 0 to 1, partly related to relatively low NIHSS scores, which may limit the generalizability of our 3-month mRS results to populations with more severe strokes or receiving more aggressive hyperacute treatment.^38^ Fourth, given that our objective was to examine how change in mRS related to long-term outcomes, we did not adjust for all possible confounding factors (aside from age/sex/NIHSS) that could also influence mortality, institutionalization, and costs, including stroke subtype, comorbidities, recurrent strokes, socioeconomic status, or adherence to rehabilitation or secondary prevention. Such factors may need to be accounted for by studies seeking to more accurately model long-term outcomes for purposes like resource allocation.

In conclusion, patients with stroke with premorbid disability have higher mortality, institutionalization, and care costs if they accumulate additional disability because of the stroke. Our study highlights the long-term outcomes expected if acute interventions are routinely withheld in patients with mild-to-moderate premorbid disability and further justifies enrolling such patients in trials or registries of acute stroke therapies.

## Acknowledgments

Dr Ganesh collected data, performed statistical analysis and interpretation, and wrote and revised the manuscript. R. Luengo-Fernandez acquired data and performed statistical analysis. S.T. Pendlebury collected data. P.M. Rothwell conceived and designed the study, provided study supervision and funding, analyzed and interpreted data, and revised the manuscript.

## Sources of Funding

The Oxford Vascular Study has been funded by the Wellcome Trust, Wolfson Foundation, and the National Institute for Health Research (NIHR) Oxford Biomedical Research Centre. P.M. Rothwell is in receipt of an NIHR Senior Investigator Award. Dr Ganesh was funded by the Rhodes Trust. We acknowledge the support of the Acute Vascular Imaging Centre, John Radcliffe Hospital, Oxford.

## Disclosures

None.

## Supplementary Material
