# Supplementary material for: Long-Term Consequences of Worsened Poststroke Status in Patients With Premorbid Disability: Implications for Treatment
Source: Stroke. 2018 Sep 10;49(10):2430–6. doi: 10.1161/STROKEAHA.118.022416 (PMC6159688; doi:10.1161/STROKEAHA.118.022416)
Supplement: Supplementary file 1 [file str-49-2430-s001.pdf]

SUPPLEMENTAL MATERIAL

SUPPLEMENTARY TABLES

Table I. Association of age, sex, and socio-economic deprivation with mild-to-moderate pre-morbid disability (mRS of 2 to 4) in patients with ischaemic stroke, excluding those with extremely severe disability, mRS=5.

	Pre-morbid mRS 2-4	
	**aOR (95%CI)**	**p>|z|**
**Age**	1.09 (1.08-1.10)	<0.0001
**Female sex**	1.61 (1.27-2.03)	<0.0001
**≥Median deprivation**	2.09 (1.64-2.67)	<0.0001
**AUC**	0.78	
**N**	1,582	
**p>|X²|**		<0.0001*

Table II – Impact of the change in mRS from pre-stroke to 3-months post-stroke (∆mRS) on 1-year and 5-year mortality, institutionalization, and death/institutionalization for 3-month survivors of ischaemic stroke with pre-morbid mRS of 2 – controlling for age, sex, and National Institutes of Health Stroke Scale (NIHSS) score at acute presentation. The institutionalization analyses exclude pre-morbidly institutionalized patients.

	1-year Mortality		5-year Mortality		5-year Institutionalization		5-year death/ institutionalization	
	**aHR(95%CI)**	**p>|z|**	**aHR(95%CI)**	**p>|z|**	**aHR(95%CI)**	**p>|z|**	**aHR(95%CI)**	**p>|z|**
**∆mRS**	0=Reference		0=Reference		0=Reference		0=Reference	
**1**	3.76 (1.26-11.2)	0.018	1.92 (1.13-3.26)	0.016	2.82 (1.27-6.29)	0.011	2.16 (1.32-3.55)	0.002
**2**	4.56 (1.23-16.9)	0.023	1.83 (0.90-3.72)	0.097	5.99 (2.33-15.4)	<0.0001	3.23 (1.70-6.17)	<0.0001
**3**	10.1 (2.25-45.9)	0.003	4.02 (1.59-10.2)	0.003	17.0 (5.61-51.6)	<0.0001	11.0 (4.66-26.2)	<0.0001
**Age**	1.02 (0.97-1.07)	0.44	1.05 (1.02-1.08)	0.001	1.06 (1.02-1.11)	0.004	1.07 (1.04-1.10)	<0.0001
**Male**	3.02 (1.28-7.11)	0.011	1.90 (1.21-2.97)	0.005	0.97 (0.50-1.87)	0.92	1.66 (1.08-2.54)	0.020
**NIHSS**	1.04 (0.97-1.12)	0.29	1.05 (1.00-1.10)	0.049	1.03 (0.97-1.09)	0.38	1.05 (1.00-1.09)	0.052
	$p>\|X^2\|$	0.0007	$p>\|X^2\|$	<0.0001	$p>\|X^2\|$	<0.0001	$p>\|X^2\|$	<0.0001
	n	187	n	187	n	184	n	184

Table III – Impact of the change in mRS from pre-stroke to 3-months post-stroke (ΔmRS) on 1-year and 5-year mortality, institutionalization, and death/institutionalization for 3-month survivors of ischaemic stroke with pre-morbid mRS of 3 – controlling for age, sex, and National Institutes of Health Stroke Scale (NIHSS) score at acute presentation. The institutionalization analyses exclude pre-morbidly institutionalized patients.

	**1-year Mortality**		**5-year Mortality**		**5-year Institutionalization**		**5-year death/ institutionalization**	
	**aHR(95%CI)**	**p>|z|**	**aHR(95%CI)**	**p>|z|**	**aHR(95%CI)**	**p>|z|**	**aHR(95%CI)**	**p>|z|**
**ΔmRS**	0=Reference		0=Reference		0=Reference		0=Reference	
**1**	2.62 (1.23-5.60)	0.013	1.32 (0.84-2.06)	0.23	2.60 (1.40-4.84)	0.003	1.88 (1.21-2.93)	0.005
**2**	5.25 (2.20-12.5)	<0.0001	3.24 (1.79-5.86)	<0.0001	3.49 (1.21-10.1)	0.021	3.36 (1.57-7.19)	0.002
**Age**	1.05 (1.01-1.10)	0.021	1.07 (1.04-1.10)	<0.0001	1.11 (1.06-1.16)	<0.0001	1.09 (1.06-1.12)	<0.0001
**Male**	2.29 (1.26-4.17)	0.007	1.74 (1.18-2.56)	0.005	1.33 (0.76-2.32)	0.31	1.66 (1.13-2.43)	0.010
**NIHSS**	1.03 (0.99-1.08)	0.14	1.02 (0.99-1.06)	0.23	1.05 (0.98-1.12)	0.18	1.05 (1.00-1.10)	0.049
	$p>\|X^2\|$	<0.0001	$p>\|X^2\|$	<0.0001	$p>\|X^2\|$	<0.0001	$p>\|X^2\|$	<0.0001
	n	176	n	176	n	154	n	154

Table IV – Impact of the change in mRS from pre-stroke to 3-months post-stroke (∆mRS) on 1-year and 5-year mortality, institutionalization, and death/institutionalization for 3-month survivors of ischaemic stroke with pre-morbid mRS of 4 – controlling for age, sex, and National Institutes of Health Stroke Scale (NIHSS) score at acute presentation. The institutionalization analyses exclude pre-morbidly institutionalized patients.

	1-year Mortality		5-year Mortality		5-year Institutionalization		5-year death/ institutionalization	
	**aHR(95%CI)**	**p>|z|**	**aHR(95%CI)**	**p>|z|**	**aHR(95%CI)**	**p>|z|**	**aHR(95%CI)**	**p>|z|**
**∆mRS**	0=Reference		0=Reference		0=Reference		0=Reference	
**1**	3.35 (1.03-10.9)	0.045	1.86 (0.72-4.79)	0.20	4.47 (1.24-16.1)	0.022	5.32 (1.72-16.5)	0.004
**Age**	1.00 (0.94-1.06)	1.00	1.04 (1.00-1.09)	0.04	1.00 (0.95-1.04)	0.92	1.02 (0.99-1.06)	0.25
**Male**	1.10 (0.32-3.75)	0.88	0.75 (0.33-1.72)	0.49	0.55 (0.19-1.64)	0.29	0.88 (0.99-1.06)	0.75
**NIHSS**	1.09 (0.98-1.21)	0.11	1.08 (1.01-1.15)	0.03	1.01 (0.91-1.12)	0.82	1.01 (0.93-1.08)	0.88
	$p>\|X^2\|$	<0.0001	$p>\|X^2\|$	0.0034	$p>\|X^2\|$	0.0770	$p>\|X^2\|$	<0.0001
	n	47	n	47	n	42	n	42

Table V – Impact of the change in mRS from pre-stroke to 3-months post-stroke (∆mRS) on 5-year health and social care costs in 3-month ischaemic stroke survivors with pre-morbid mRS of 2-4.

	**All**		**Pre-morbid mRS of 2**		**Pre-morbid mRS of 3**		**Pre-morbid mRS of 4**	
	**Margins ($, 95%CI)**	**p>|z|**	**Margins ($)**	**p>|z|**	**Margins ($)**	**p>|z|**	**Margins ($)**	**p>|z|**
**∆mRS**	0=Reference		0=Reference		0=Reference		0=Reference	
1	623 (-9,693-10,940)	0.91	11,013 (101-21,925)	0.048	-8,372 (-24,567-7,824)	0.31	21,724 (-37,880-81,327)	0.48
2	24,918 (571-49,265)	0.015	53,377 (11,936-94,818)	0.012	7,151 (-23,685-37,987)	0.65	N/A	
3	39,699 (-24,578-103,977)	0.086	72,057 (-14,590-158,705)	0.103	N/A		N/A	
**Age**	446 (-66-958)	0.088	681 (22-1,339)	0.043	1,062 (94-2,029)	0.032	-1,722 (-3,794-350)	0.10
**Male**	-8,700 (-19,178-1,777)	0.10	-7,844 (-19,894-4,205)	0.20	-8,509 (-24,784-7,767)	0.31	-30,702 (-76,956-15,552)	0.19
**NIHSS**	-336 (-1,446-773)	0.55	-746 (-2,308-815)	0.35	-757 (-2,394-880)	0.37	-722 (-4,256-2,811)	0.69
	n	410	n	187	n	176	n	47

Table VI – Impact of the change in mRS from pre-stroke to 3-months post-stroke (∆mRS) on 5-year health and social care costs in 3-month ischaemic stroke survivors with pre-morbid mRS of 2-4, excluding pre-morbidly institutionalized patients (n=34) and censored patients (n=29, 2 of whom were already institutionalized).

	**All**		**Pre-morbid mRS of 2**		**Pre-morbid mRS of 3**		**Pre-morbid mRS of 4**	
	**Margins ($, 95%CI)**	**p>|z|**	**Margins ($)**	**p>|z|**	**Margins ($)**	**p>|z|**	**Margins ($)**	**p>|z|**
**∆mRS**	0=Reference		0=Reference		0=Reference		0=Reference	
1	5,731 (-5,822-16,564)	0.35	10,387 (-1,266-22,040)	0.081	-779 (-18,148-16,590)	0.93	39,119 (-41,230-119,468)	0.34
2	38,014 (5,628-70,401)	0.021	55,619 (11,683-99,555)	0.013	20,928 (-20,911-62,766)	0.33	N/A	
3	54,762 (-28,758-138,281)	0.20	73,492 (-17,802-164,786)	0.12	N/A		N/A	
**Age**	216 (-299-731)	0.41	732 (23.9-1,440)	0.043	611 (-349-1,571)	0.21	-1,760 (-3,937-418)	0.11
**Male**	-8,874 (-19,857-2,109)	0.11	-9,473 (-22,432-3,485)	0.15	-7,624 (-24,407-9,159)	0.37	-35,849 (-89,902-18,204)	0.19
**NIHSS**	-991 (-2,260-279)	0.13	-783 (-2,492-926)	0.37	-1,455 (-3,321-410)	0.13	-2,218 (-5,909-1,472)	0.24
	n	355	n	167	n	147	n	41

SUPPLEMENTARY FIGURES

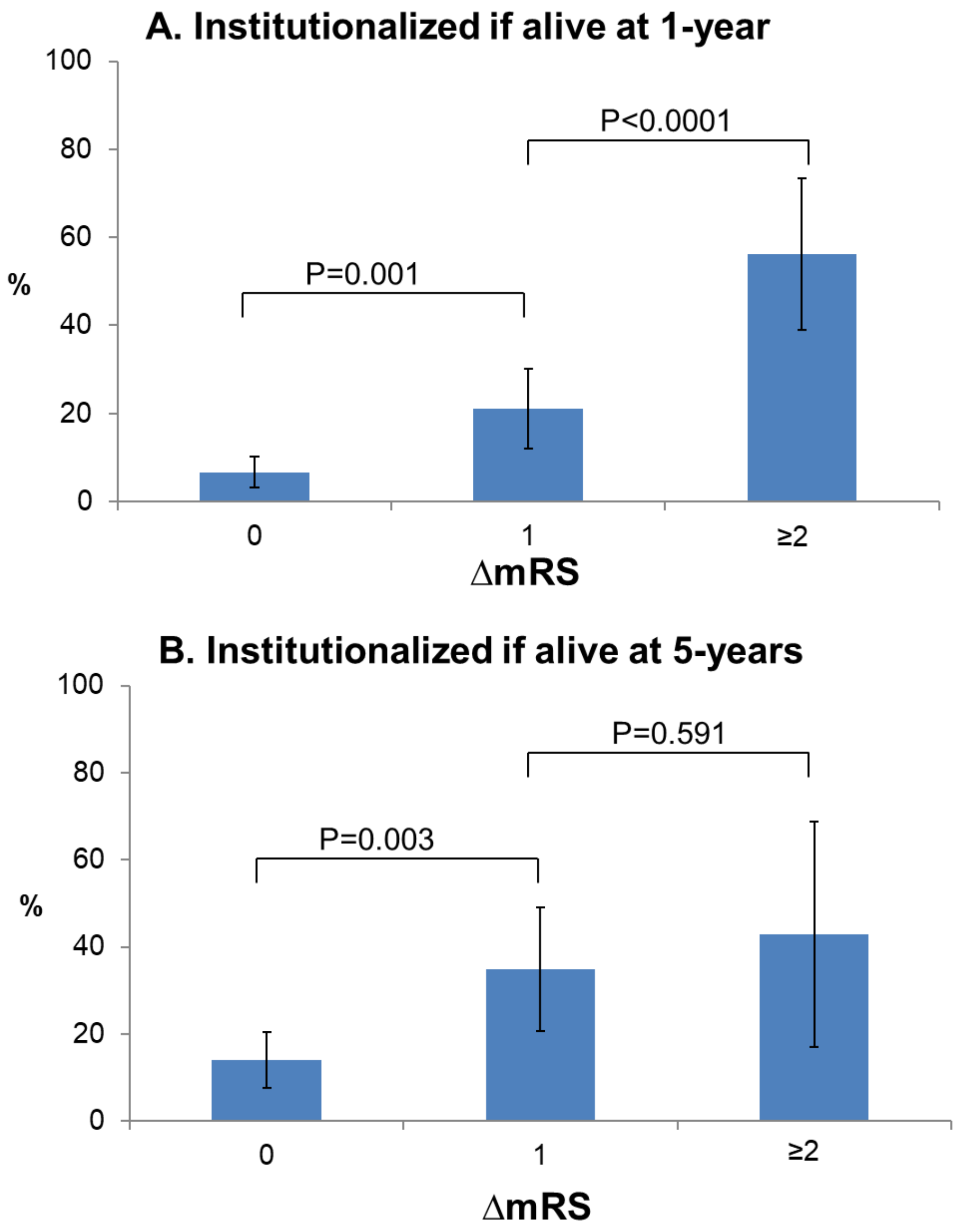

Figure I. 1-year and 5-year institutionalization outcomes in survivors of ischaemic stroke with pre-morbid mRS of 2-4, stratified by the change in mRS between pre-stroke and 3-months post-stroke (ΔmRS). The graphs show the proportion of 3-month survivors, also alive at (A) 1-year, who were institutionalized by 1-year post-stroke, and (B) also alive at 5-years, who were institutionalized by 5-years post-stroke. Institutionalization was defined as admission to a nursing or residential care home. The P-values from chi-squared tests for differences between mRS grades are indicated. Bars represent 95% confidence intervals.

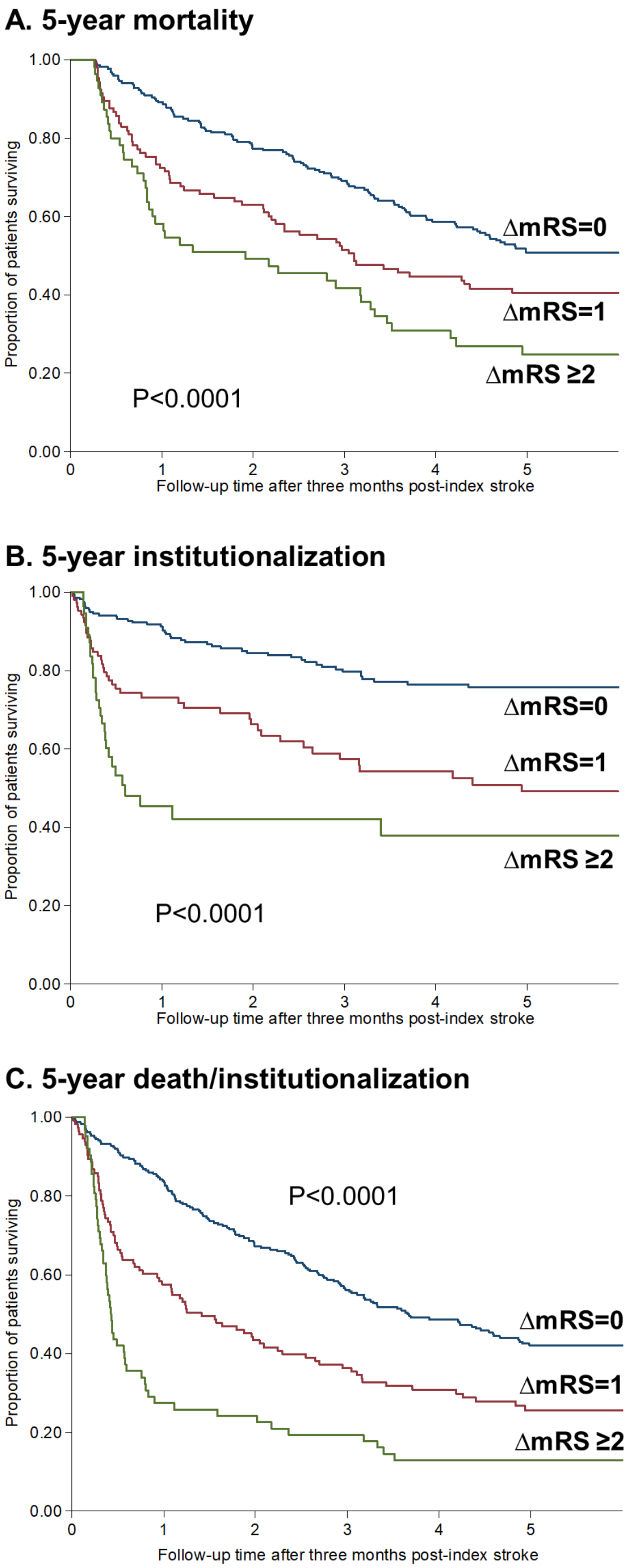

Figure II. Kaplan-Meier survival curves after index stroke for 3-month survivors with pre-morbid mRS of 2-4, stratified by the change in mRS between pre-stroke and 3-months post-stroke (ΔmRS), for (A) 5-year mortality, (B) 5-year institutionalization, and (C) 5-year mortality/institutionalization. Since only 3-month survivors with a pre-morbid mRS of 2 could have a ΔmRS of 3, we combined them with patients with a pre-morbid mRS of 3 who had a ΔmRS of 2 ("ΔmRS≥2"). P-values from log-rank test for each group are shown.
